# A Journey towards Understanding the Molecular Pathology and Developing Therapies for Lysosomal Storage Disorders

**DOI:** 10.3390/cells11010036

**Published:** 2021-12-23

**Authors:** Ritva Tikkanen

**Affiliations:** Medical Faculty, Institute of Biochemistry, University of Giessen, Friedrichstrasse 24, 35392 Giessen, Germany; Ritva.Tikkanen@biochemie.med.uni-giessen.de

Lysosomal storage disorders (LSDs) are rare, monogenic diseases characterized by aberrant lysosomes with storage material. These diseases often manifest as neurodegeneration and developmental delay, together with further defects such as organomegaly, vision impairment and skeletomuscular problems. Many LSDs are also associated with a reduced life span, but life expectancy is highly variable between the different LSDs, ranging from very early death to near-normal life expectancy. Most LSDs result from the deficiency of a single enzyme, whereas others are caused by mutations in non-enzymatic proteins. The molecular mechanisms and cellular pathology of these diseases have been subject to intensive research for decades, but for many of these diseases, no approved therapy options are available. However, ever more preclinical studies on novel therapies such as gene therapy, chaperone therapy, substrate reduction and enzyme replacement therapy (ERT) have emerged in the past few years.

The purpose of this Special Issue (SI) is to summarize our current understanding of the disease pathogenesis and molecular mechanisms of LSDs, and to explore therapeutic strategies that can be used in LSDs. In addition, this SI addresses the involvement of various cellular pathways such as autophagy and organelle crosstalk in the pathogenesis of LSDs. Novel treatment concepts, natural history studies, diagnostic tools and disease biomarkers are also a subject of this SI, which contains both timely review articles and original research papers.

Hematopoietic cell transplant (HCT) and bone marrow transplantation were treatment options explored many years ago for several LSDs, with varying success. In their review article, Naumchik et al. summarize the history of HCT in glycoprotein storage disorders and compare its success with ERT in diseases for which both options have been explored [1]. They conclude that HCT should be pursued early in life, but the efficiency may still vary largely, and some diseases may be poor candidates for HCT. However, for a specific group of LSDs, HCT, when carried out very early, may still provide a valid therapy option [1].

Since early diagnosis is very important for many treatment options, especially in diseases that manifest very early in life, newborn screening (NBS) programs and diagnostics based on genomics tools, such as sequencing, will facilitate an early identification of LSD patients. La Cognata et al. provide an update on worldwide NBS programs in LSDs, and on the importance of the use of modern genomics tools for diagnostic and research purposes [2]. With the emergence of multinational NBS programs and platforms, such as MetabERN (European Reference Network for Hereditary Metabolic Disorders), the awareness of clinicians regarding tools and treatment options of rare diseases will be enhanced [2]. In the long run, this will hopefully provide earlier diagnoses and access to treatment options for many more patients worldwide.

LSDs frequently present as multi-system disorders that require the management of symptoms that are present in various organ systems. Yim et al. provide an overview and clinical recommendations for the management of cardiomyopathy in Fabry disease (FD), an X-linked LSD [3]. They stress that FD should be considered as a potential diagnosis in patients that show a thickening of the left ventricular wall that cannot be otherwise explained [3], since as much as 4 to 12% of such cases may be caused by FD [4,5,6]. As treatment options including ERT, substrate reduction and oral chaperones are available for FD, the early identification of FD in patients is of particular importance.

Mucopolysaccharidoses (MPS) are a group of LSDs that affect the degradation of glycosaminoglycans (GAGs) in lysosomes. Three articles in this SI have been dedicated to various aspects of MPS. Hampe et al. summarize the natural history and molecular pathology in MPS type I [7]. In accordance with the above studies, they also stress the importance of the early diagnosis of MPS I, so that potentially fatal complications of the disease can be managed and avoided. De Pasquale et al. provide an overview of the cathepsin proteases in MPS [8]. Pathogenic variants of cathepsins A, D, F and K are the genetic cause of some LSDs, but cathepsins also play a role in numerous other diseases [9]. In MPS, abnormal activity and regulation of cathepsins are involved in the skeletal and neuronal pathology, and inhibition of selected cathepsins may thus provide a novel treatment option in MPS, which should be explored in preclinical and clinical studies [8].

Lung manifestations are a typical feature of many LSDs. Paget et al. explored the importance of surfactant function in a mouse model for MPS IIIA [10]. They show that altered amounts of heparan sulphate, specific lipids and surfactant components are observed in pulmonary tissue and bronchoalveolar lavage fluid of this mouse model [10]. Thus, the lung manifestations in MPS IIIA may be exacerbated by the altered composition of lung surfactant.

Although the primary organelle dysfunction in LSD is the deficiency of lysosomal function, an impairment of the function of further organelles, especially mitochondria, has been detected in many LSDs. In their review article, Kuk et al. summarize the current knowledge on the crosstalk between mitochondrial and lysosomes and suggest that this crosstalk may provide a novel means for treatment of LSDs [11].

Dysfunctional mitochondria are removed by autophagy, and defects in autophagy are commonly found in LSDs. Petcherski et al. carried out a high-content screen to identify small molecules that modify autophagy and could this provide a potential pharmacological treatment for the juvenile form of neuronal ceroid lipofuscinosis (jNCL) [12]. They identified several cellular processes, such as calcium signaling and the mevalonate pathway, the modulation of which resulted in an improvement of the autophagy defects and, in some cases, ameliorated the lysosomal dysfunction. This study thus suggests that drugs that target these pathways may be novel candidates for the treatment of LSD [12].

Ever more preclinical and clinical studies aiming at treatment for LSD are currently being conducted. For monitoring the effect of such therapy trials, biomarkers that can provide a quantitative assessment of the treatment effect are required. Percival et al. describe a novel strategy for the identification of new biomarkers for GM1 type 2 gangliosidosis, with a nuclear magnetic resonance (NMR)-linked metabolomics strategy [13]. The development of such methods for further LSD is of high importance, as they also facilitate diagnosis and may identify novel metabolic targets for therapy interventions.

Many LSDs are based on the defects of single enzymes, and the measurement of enzyme activity is an important part of the diagnosis of these LSDs, but can also be used as a biomarker for clinical studies. However, the choice of material used for the enzyme activity measurement is important for obtaining reliable data. Strobel et al. analyzed the reliability of enzyme activity measurements in metachromatic leukodystrophy and gangliosidoses [14]. They show that the enzyme activities in different leucocyte subpopulations may vary considerably. In addition, the preparation of the sample (cell lysate) for the activity measurement is also an important factor that affects the measurements [14]. Therefore, the careful optimization of the protocols is a prerequisite for accurate activity measurements.

Gene therapy has been viewed as a promising treatment option for LSD. For a recent summary on gene therapy approaches in LSD, the readers are referred to the recent review by Massaro et al. (not part of our SI) [15]. Most of the gene therapy approaches for LSDs are based on adeno-associated virus (AAV), typically an AAV serotype 9 that is capable of infecting the brain. However, a major drawback of AAV9 is that it only poorly infects cultured human cell lines. Since potency assays for the demonstration of the efficiency of the gene therapy vector are requested by regulatory authorities, an assay that can be carried out in standard cell lines is desirable. The paper from my own group, by Banning et al., shows that the knockout of the CMP-sialic acid transporter SCL35A1 in human embryonic kidney (HEK) cells results in an efficient transduction with AAV9, providing a novel tool for the development of potency assays [16].

Animal models are of vital importance for studies of LSD pathology and the development of treatments. However, the use of some animal models may be complicated by genetic differences to humans. Gaucher disease (GD) is caused by the deficiency of the lysosomal acid β-glucocerebrosidase (GCase). In *Drosophila melanogaster*, the fruit fly, there are two gene orthologs, of which *GBA1b* was shown to be equivalent to GCase [17]. Cabasso et al. characterized the function of the second gene ortholog, *GBA1a*, which was shown to be involved in the maturation of the mid-gut during larval development [18]. The authors show that a mutant version of the protein encoded by *GBA1a* activated the unfolded protein response (UPR), further demonstrating the function of the gene product in the regulation of cell survival [18].

Taken together, the present SI provides a collection of original articles and reviews that address various features of LSD. Collectively, these articles provide an overview of the recent developments in the pathology, management and treatment of various LSDs. As this field is continuously developing, we look forward to future research on this important topic.

## References

[1] Naumchik B.M., Gupta A., Flanagan-Steet H., Steet R.A., Cathey S.S., Orchard P.J., Lund T.C. (2020). The Role of Hematopoietic Cell Transplant in the Glycoprotein Diseases. Cells.

[2] La Cognata V., Guarnaccia M., Polizzi A., Ruggieri M., Cavallaro S. (2020). Highlights on Genomics Applications for Lysosomal Storage Diseases. Cells.

[3] Yim J., Yau O., Yeung D.F., Tsang T.S.M. (2021). Fabry Cardiomyopathy: Current Practice and Future Directions. Cells.

[4] Linhart A., Germain D.P., Olivotto I., Akhtar M.M., Anastasakis A., Hughes D., Namdar M., Pieroni M., Hagege A., Cecchi F. (2020). An expert consensus document on the management of cardiovascular manifestations of Fabry disease. Eur. J. Heart Fail..

[5] Sachdev B., Takenaka T., Teraguchi H., Tei C., Lee P., McKenna W.J., Elliott P.M. (2002). Prevalence of Anderson-Fabry disease in male patients with late onset hypertrophic cardiomyopathy. Circulation.

[6] Maron M.S., Xin W., Sims K.B., Butler R., Haas T.S., Rowin E.J., Desnick R.J., Maron B.J. (2018). Identification of Fabry Disease in a Tertiary Referral Cohort of Patients with Hypertrophic Cardiomyopathy. Am. J. Med..

[7] Hampe C.S., Eisengart J.B., Lund T.C., Orchard P.J., Swietlicka M., Wesley J., McIvor R.S. (2020). Mucopolysaccharidosis Type I: A Review of the Natural History and Molecular Pathology. Cells.

[8] De Pasquale V., Moles A., Pavone L.M. (2020). Cathepsins in the Pathophysiology of Mucopolysaccharidoses: New Perspectives for Therapy. Cells.

[9] Patel S., Homaei A., El-Seedi H.R., Akhtar N. (2018). Cathepsins: Proteases that are vital for survival but can also be fatal. Biomed. Pharm..

[10] Paget T.L., Parkinson-Lawrence E.J., Trim P.J., Autilio C., Panchal M.H., Koster G., Echaide M., Snel M.F., Postle A.D., Morrison J.L. (2021). Increased Alveolar Heparan Sulphate and Reduced Pulmonary Surfactant Amount and Function in the Mucopolysaccharidosis IIIA Mouse. Cells.

[11] Kuk M.U., Lee Y.H., Kim J.W., Hwang S.Y., Park J.T., Park S.C. (2021). Potential Treatment of Lysosomal Storage Disease through Modulation of the Mitochondrial-Lysosomal Axis. Cells.

[12] Petcherski A., Chandrachud U., Butz E.S., Klein M.C., Zhao W.N., Reis S.A., Haggarty S.J., Ruonala M.O., Cotman S.L. (2019). An Autophagy Modifier Screen Identifies Small Molecules Capable of Reducing Autophagosome Accumulation in a Model of CLN3-Mediated Neurodegeneration. Cells.

[13] Percival B.C., Latour Y.L., Tifft C.J., Grootveld M. (2021). Rapid Identification of New Biomarkers for the Classification of GM1 Type 2 Gangliosidosis Using an Unbiased (1)H NMR-Linked Metabolomics Strategy. Cells.

[14] Strobel S., Hesse N., Santhanakumaran V., Groeschel S., Bruchelt G., Krageloh-Mann I., Bohringer J. (2020). Optimization of Enzyme Essays to Enhance Reliability of Activity Measurements in Leukocyte Lysates for the Diagnosis of Metachromatic Leukodystrophy and Gangliosidoses. Cells.

[15] Massaro G., Geard A.F., Liu W., Coombe-Tennant O., Waddington S.N., Baruteau J., Gissen P., Rahim A.A. (2021). Gene Therapy for Lysosomal Storage Disorders: Ongoing Studies and Clinical Development. Biomolecules.

[16] Banning A., Zakrzewicz A., Chen X., Gray S.J., Tikkanen R. (2021). Knockout of the CMP-Sialic Acid Transporter SLC35A1 in Human Cell Lines Increases Transduction Efficiency of Adeno-Associated Virus 9: Implications for Gene Therapy Potency Assays. Cells.

[17] Kinghorn K.J., Gronke S., Castillo-Quan J.I., Woodling N.S., Li L., Sirka E., Gegg M., Mills K., Hardy J., Bjedov I. (2016). A Drosophila Model of Neuronopathic Gaucher Disease Demonstrates Lysosomal-Autophagic Defects and Altered mTOR Signalling and Is Functionally Rescued by Rapamycin. J. Neurosci..

[18] Cabasso O., Paul S., Maor G., Pasmanik-Chor M., Kallemeijn W., Aerts J., Horowitz M. (2021). The Uncovered Function of the Drosophila GBA1a-Encoded Protein. Cells.

